# Valorization of Teak Leaf Agricultural Waste: Impact of Drying Technique and Production Scale on Extract Quality

**DOI:** 10.1021/acsomega.6c00281

**Published:** 2026-06-11

**Authors:** Natthawadee Tibkawin, Nichapa Buasumrit, Panatpong Boonnoun, Sukunya Ross, Gareth Ross, Jarupa Viyoch, Pensri Charoensit

**Affiliations:** † Department of Pharmaceutical Technology, Faculty of Pharmaceutical Sciences and Center of Excellence for Innovation in Chemistry (PERCH-CIC), 567889 Naresuan University, Phitsanulok 65000, Thailand; ‡ Department of Industrial Engineering, Chemical Engineering Program, Faculty of Engineering, 59212 Naresuan University, Phitsanulok 65000, Thailand; § Department of Chemistry, Center of Excellence in Biomaterials, Faculty of Science, 59212 Naresuan University, Phitsanulok 65000, Thailand

## Abstract

This study investigates how drying technique and production scale govern the quality, structure, and functionality of teak leaf extracts derived from an abundant agricultural byproduct. Freeze-drying (FD) and spray-drying (SD) were applied at laboratory and pilot scales, and the resulting extracts were evaluated for physicochemical properties, bioactive composition, antioxidant activity, powder flowability, and thermal behavior. Across both leaf maturities, FD preserved higher total phenolic and flavonoid contents and yielded extracts with higher antioxidant activity (DPPH IC_50_: 20–25 μg/mL) than SD-derived extracts (DPPH IC_50_: 30–43 μg/mL), while all samples exhibited strong radical-scavenging capacity overall (DPPH IC_50_: 19.93–42.61 μg/mL), underscoring the high intrinsic antioxidant potential of teak leaf extracts. Thermal analysis revealed that FD extracts formed more crystalline and energetically stable matrices, whereas SD produced predominantly amorphous structures with improved thermal dryness. At the laboratory scale, SD powders exhibited low moisture content, good flowability, and uniform spherical morphology, while FD powders showed porous, irregular structures associated with reduced flow. Importantly, pilot-scale processing altered both thermal stability and phenolic retention, particularly in young leaf extracts, indicating that laboratory-scale drying behavior cannot be directly extrapolated to larger-scale production. These results demonstrate that drying technique and production scale jointly influence the structure–property relationships of teak leaf extracts. The findings support tailored use of FD and SD extracts according to formulation requirements and highlight the potential of teak leaves as a sustainable source of natural antioxidants and colorants for food, cosmetic, and pharmaceutical applications.

## Graphical abstract

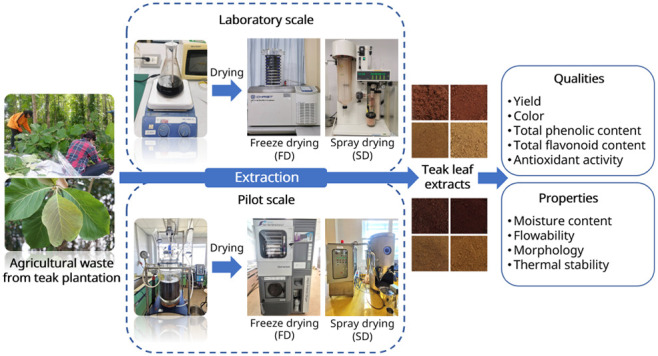

## 1 Introduction

Agricultural industries generate substantial quantities of waste and byproducts, including crop residues and animal waste. These materials are often underutilized or disposed of inappropriately, which leads to significant environmental challenges. Improper management of agricultural waste has been associated with soil degradation, water and air pollution, loss of biodiversity, and increased greenhouse gas emissions. Collectively, these effects contribute to climate change and pose serious risks to public health.^1,2^ In line with circular economy and green chemistry principles, the valorization of agricultural residues into high-value products represents an environmentally responsible strategy for resource management. Notable examples include the extraction of plant polysaccharides from fruit and vegetable processing residues,^3^ the production of biodegradable foam from sugarcane bagasse,^4^ and the fabrication of biodegradable nursery bags from spent coffee grounds.^5^ Turning these underused residues into valuable products not only reduces waste but also supports sustainable economic development, particularly in regions where these residues are abundant.

Teak (*Tectona grandis* *L*. f.) is one of the most valuable tropical hardwoods, renowned for its high-quality timber and economic importance. It is a key component of forestry industries in many tropical countries and is widely distributed in India, Myanmar, Laos, Thailand, and other parts of Southeast Asia.^6^ Teak plantations produce substantial leaf litter during periodic shedding and maintenance; this material is often undervalued and mismanaged, and in dry seasons or under certain practices (e.g., burning), it becomes a source of air pollution, fire risk, and waste accumulation in plantations and surrounding communities.^7^ While teak leaves have long been used in folk medicine to alleviate inflammation and related disorders, they have recently been gaining attention for conversion into eco-friendly materials. These include natural pigments and dyes,^8,9^ biochar,^10^ and packaging materials^11^ because of their chemical and physical behavior. Teak leaves are rich in bioactive phytochemicals such as flavonoids, tannins, anthraquinones, and anthocyanins, which provide antioxidant activity and pigment potential.^12^ These properties make teak leaf extracts promising for sustainable applications in textiles, cosmetics, packaging, and other material systems requiring natural colorants with functional benefits.

Drying is a fundamental step in converting plant extracts from liquid to stable powders suitable for storage, formulation, and transport. Among drying methods, spray drying and freeze-drying are widely used for preserving plant-based bioactives, but they differ substantially in mechanisms, cost, throughput, and effects on sensitive compounds.^13,14^ Spray drying can deliver high industrial productivity and lower operating costs, but exposure to high temperatures and rapid drying can lead to the degradation of thermolabile (heat-sensitive) molecules and the loss of volatile compounds.^15^ Freeze-drying (lyophilization), by contrast, preserves such compounds more effectively by sublimating moisture under low temperatures, although at the expense of longer processing times, higher energy use, and greater cost.^16^ Therefore, the choice of drying method can strongly influence not only the yield and physical and functional quality of powders (e.g., flavonoid/polyphenol retention, antioxidant activity, solubility, and color) but also the sustainability and economics of production.

Previous studies, including our own, have largely examined teak leaf valorization at the laboratory scale, with an emphasis on solvent selection, extraction techniques, and their effects on bioactive yield and antioxidant activity.^8,9,17,18^ In contrast, investigations at the pilot scale remain limited, and to date, no study has systematically evaluated the combined effects of drying technique and production scale on the physicochemical and bioactive properties of teak leaf extracts. To address this gap, the present work examines freeze-drying and spray-drying applied at both laboratory and pilot scales, with a focus on how these processing variables shape extract composition, thermal behavior, and powder functionality. By integrating compositional analysis with physical and structural characterization, this study establishes process–property relationships that support scale-appropriate strategies for converting teak leaf waste into value-added natural colorants and antioxidants for sustainable food, cosmetic, and pharmaceutical applications.

## 2 Experimental Section

### 2.1 Materails

All chemical reagents used in this work were of analytical grade. Ethanol was purchased from Labscan Asia Co. Ltd. (Bangkok, Thailand). DPPH (2,2-diphenyl-1-picrylhydrazyl), ABTS (2,2′-azino-bis­(3-ethylbenzothiazoline-6-sulfonic acid), l-ascorbic acid (99% purity), Trolox (6-hydroxy-2,5,7,8-tetramethylchroman-2-carboxylic acid), rutin hydrate, and gallic acid were provided by Sigma–Aldrich (St. Louis, MO, USA). Potassium persulfate and Folin–Ciocalteu’s phenol reagent were obtained from Merck (Darmstadt, Germany), sodium carbonate (Na_2_CO_3_) from Ajax Finechem (Victoria, Australia), and aluminum chloride hexahydrate (AlCl_3_ 6H_2_O) from Kemaus (New South Wales, Australia).

### 2.2 Preparation of Teak Leaves Materials

Teak leaves were collected from Phichit province, Thailand. The young reddish-green leaves, which are the two leaves at the top of the branch, while the mature green leaves, which are the leaves below, as shown in Figure 1, were harvested in June. Teak leaves were also authenticated by Dr. Anchalee Nuammee of the Department of Biology, Faculty of Science, Naresuan University (voucher specimen number: 06083). The leaves were washed and dried in a hot air oven at 60 °C for 24 h, then ground into powdered leaves using a blender and sieved by a horizontal vibratory sieve shaker (AS 200, Retsch) comprising four sieves with opening sizes of 250, 180, 150, and 75 μm.

**Figure 1:**
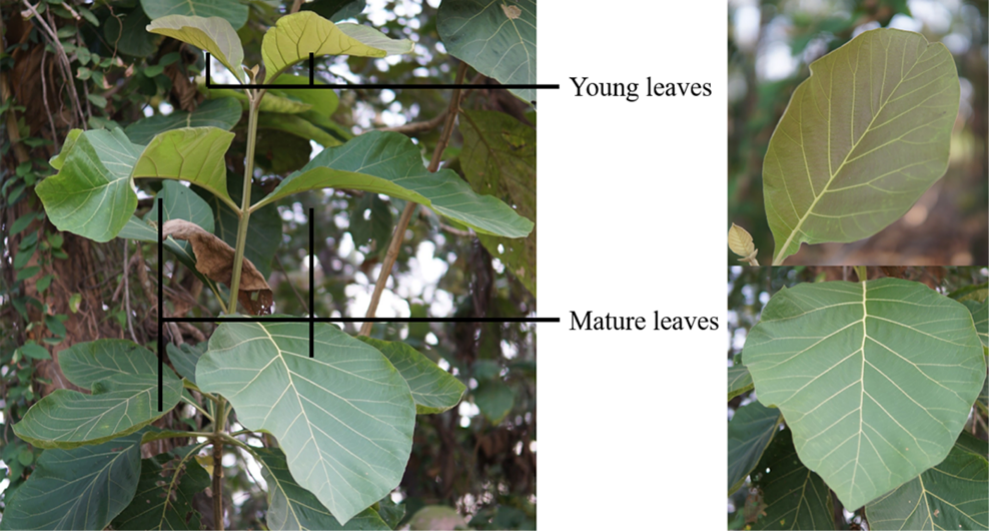
The image shows the position and appearance of young and mature teak leaves.

### 2.3 Preparation of Teak Leaf Extracts at Laboratory and Pilot Scale

Eight different extracts were prepared from young and mature teak leaves following the procedure described in Thailand Petty Patent No. 15809.^19^ Briefly, 100 g of powdered leaves were extracted with 1 L of 50% (v/v) ethanol using the decoction method in a 2 L flat-bottom flask. The mixture was heated at approximately 70 °C on a hot plate stirrer for 3 h and subjected to two extraction cycles. The extracts were filtered through Whatman No. 1 filter paper under suction, and ethanol was removed under reduced pressure using a rotary evaporator (Rotavapor R-210, Büchi, England). The resulting crude extracts were divided equally into two portions: one for freeze-drying and the other for spray-drying, and stored at 4 °C until further processing.

For freeze-drying, the extracts were prefrozen at −80 °C for 24 h, then lyophilized using a freeze-dryer (Gamma 2–16 LSC, Christ, Osterode, Germany) at a condenser temperature of −70 °C and a vacuum pressure of 0.12 mbar. The freeze-drying process was set at a shelf temperature of 10 °C for 68 h for the primary drying step, followed by 25 °C for 4 h for the secondary drying step. The freeze-dried powders were stored in amber glass bottles at −20 °C until analysis.

For spray-drying, the extracts were processed using a mini spray dryer (B-290, Büchi Labortechnik AG, Flawil, Switzerland) equipped with a two-fluid nozzle (particle size 2–25 μm). Operating parameters were as follows: aspirator rate 100% (35 m^3^/h), atomizing air flow 473 L/h (rotameter 40 mm) with cocurrent flow, inlet temperature 170 °C, and feed rate 20% (6 mL/min) to maintain an outlet temperature of 110 °C. Upon completion, samples were collected after the inlet temperature cooled below 50 °C and stored in amber glass bottles at −20 °C until analysis.

Pilot-scale extraction was conducted under conditions adapted from the laboratory-scale process. A 50 L triple-wall glass reactor (GR-50CE, Greatwall, Siam Intercrop, Thailand), equipped with a low-temperature stirring reaction bath (SIT109, DHJF Series, Greatwall, Siam Intercrop, Thailand), was used for scale-up experiments. Three kilograms of powdered teak leaves were extracted with 30 L of 50% (v/v) ethanol at 70 °C for 3 h in two extraction cycles. Continuous agitation was maintained with a propeller stirrer at 120 rpm to ensure uniform solvent circulation. The mixtures were filtered through Whatman No. 1 filter paper using a 50 L glass vacuum filtration unit (ZF-50L, Xingyang Kori Instrument Factory, China), and ethanol was removed from the filtrates using a rotary evaporator (EXR-1020, Lanphan, China).

Pilot-scale drying was then performed using the concentrated extracts. The first half of the extracts was prefrozen at −80 °C using an upright ultralow temperature freezer for 24 h and dried using a 35 L pilot freeze-dryer (Genesis SQ EL-85, Virtis, SP Scientific, Warminster, PA, USA) under the conditions adapted from the laboratory scale, with a condenser temperature of −70 °C and a chamber pressure of 57 mTorr. The freeze-drying process was conducted at a shelf temperature of 0 °C for 12 h, followed by 30 °C for 72 h.

The remaining half of the extracts was spray-dried using a pilot-scale spray dryer (SD-01, HVAC Engineering Corporation Ltd., Thailand) equipped with a two-fluid nozzle. Operating conditions were as follows: feed rate 0.75 L/h (12.5 mL/min; pump scale 0.5), inlet temperature 170 °C, and outlet temperature 110 °C. After drying, the powders were collected once the inlet temperature had decreased below 50 °C and stored in amber glass bottles at −20 °C until further analysis.

The percentage yield of the freeze-dried and spray-dried products was calculated using eq 1:

1 $$\mathrm{\%Yield}=\frac{P}{R}\times 100$$

where *P* is the amount of teak leaf extracts (g), and *R* is the amount of teak leaf powder (g).

### 2.4 Color Determination of Teak Leaf Extracts

The color values of the pigment were measured using a colorimeter (ColorQuest XE, Hunterlab, USA) in reflectance specular-excluded mode. Approximately 5 g of the teak leaf extract was placed in the sample container. The values of L* (darkness/lightness), a* (greenness/redness), and b* (blueness/yellowness) were measured in the CIELab color space.

### 2.5 Determination of Total Flavonoid Content (TFC)

The total flavonoid content of teak leaf extracts was determined according to the method of Stankovic (2011) and Tibkawin (2022).^9,20^ Each extract was dissolved in 50% (v/v) ethanol to a concentration of 1 mg/mL. A 100 μL aliquot of the sample was mixed with 100 μL of 2% (w/v) AlCl_3_ 6H_2_O in methanol and incubated for 1 h at room temperature. Absorbance was recorded at 415 nm using a microplate reader. All measurements were performed in triplicate. Total flavonoid content was calculated from a rutin calibration curve (10–120 μg/mL) and expressed as mg rutin equivalent (RU) per g of extract.

### 2.6 Determination of Total Phenolic Content (TPC)

The total phenolic content of teak leaf extracts was determined using the Folin-Ciocalteu method.^9,21^ Each extract was dissolved in 50% (v/v) ethanol to a concentration of 10 mg/mL. A reaction mixture consisting of 178 μL of purified water, 10 μL of 20% (w/v) Na_2_CO_3_, 10 μL of Folin–Ciocalteu reagent, and 2 μL of sample was prepared in triplicate. After incubation for 30 min at 25 °C, absorbance was measured at 765 nm using a microplate reader. Total phenolic content was calculated from a gallic acid calibration curve (200–1,000 μg/mL) and expressed as mg gallic acid equivalent (GAE) per g of extract.

### 2.7 Determination of Antioxidant Activity (AA)

The antioxidant activity of teak leaf extracts was evaluated based on their 2,2-diphenyl-1-picrylhydrazyl (DPPH) radical scavenging capacity. Extracts were dissolved in 50% (v/v) ethanol to prepare final concentrations ranging from 0.1 to 1,000 μg/mL. After incubation in the dark for 30 min at room temperature, absorbance was measured at 515 nm using a microplate reader.

The ABTS radical scavenging activity was also modified from Yan F (2018).^22^ Extracts were dissolved in 50% (v/v) ethanol to prepare final concentrations ranging from 0.02 to 200 μg/mL. The absorbance of the mixture was immediately measured at 734 nm using a microplate reader after a 6-min incubation in the dark at room temperature.

The percentage inhibition of both DPPH and ABTS assays was calculated using eq 2:

2 $$\%\mathrm{inhibition}=\frac{\left(A_{\mathrm{control}}-A_{\mathrm{sample}}\right)}{A_{\mathrm{control}}}\times 100$$

where A control is the absorbance of the control solution (DPPH or ABTS without sample), and A sample is the absorbance of the reaction mixture containing the extract. The half-maximal inhibitory concentration (IC_50_), representing the extract concentration required to scavenge 50% of DPPH and ABTS radicals, was determined from the dose–response curve using nonlinear regression analysis.

### 2.8 Determination of Moisture Content of Teak Leaf Extracts

Moisture content was determined using a moisture analyzer (MA37, Sartorius, Göttingen, Germany). The accurately 2 g of the sample was evenly spread on an aluminum tray (ca.10 cm in diameter) and heated at 105 °C. The moisture percentage (%M) was recorded when the rate of change was less than 0.05% per 30 s (equivalent to 1 mg/min).

### 2.9 Measurement of Flowability of Teak Leaf Extracts

The flowability of the extract powders was evaluated using the angle of repose method according to the United States Pharmacopeia (USP) <1174>.^23^ Approximately 5 g of powder was allowed to flow through a funnel positioned 5 cm above a flat surface to form a conical pile. The height (*h*) and base diameter (*d*) of the pile were measured, and the angle of repose (α) was calculated using eq 3:

3 $$\tan\left(\alpha \right)=\frac{h}{0.5d}$$

Flowability was classified based on α, by Carr* index as follows: 25–30°, excellent; 31–35°, good; 36–40°, fair–aid not needed; 41–45°, passable–may hang up; 46–55°, poor–must agitate, vibrate; 56–65°, very poor; and >66°, very, very poor.

### 2.10 Characterization Analysis of Teak Leaf Extracts

#### 2.10.1 DSC Analysis

Glass transition temperature (Tg) and melting temperature (Tm) were determined using a differential scanning calorimeter (DSC1 Star System, Mettler Toledo, OH, USA), following the method of Liu et al. (2018).^24^ Samples (2–5 mg) were sealed in aluminum pans, with an empty pan as a reference. Each sample was heated from 25 to 200 °C at 10 °C/min, cooled to 25 °C, and reheated to 300 °C at the same rate under a nitrogen flow of 50 mL/min. The Tg was obtained from the midpoint of the heat capacity change during the second heating cycle. Data analysis, including baseline correction and thermogram processing, was performed using STARe software (v12.10, Mettler Toledo, OH, USA). The onset and peak temperatures, and mean normalized enthalpy (J/g) corresponding to the endothermic peak of teak leaf extracts were recorded.

#### 2.10.2 Scanning Electron Microscope (SEM)

The morphologies and microstructures of teak leaf extracts were observed by field emission scanning electron microscopy (FESEM) (Apero S, Thermo Fisher, USA) at an accelerating voltage of 5 kV. The samples were placed on stubs and coated with gold in argon using an Edwards Sputter Coater S150A instrument to prevent a charging effect under the electron beam.

## 3 Results and Discussion

### 3.1 The Effect of Production Scale, Drying Method and Leaf Maturity on the Extraction Yield and Color Appearance of Teak Leaf Extracts

The drying method influenced the extraction yield of teak leaf extracts at both laboratory and pilot scales. At the laboratory scale, the freeze-dried (FD) young leaf extract showed a slightly higher yield (22.90%) than the spray-dried (SD) extract (21.35%), while for mature leaves, the FD extract (17.53%) yielded substantially more than the SD extract (13.09%) (Table 1). These trends were consistent at the pilot scale, where FD extracts similarly provided higher recovery than their SD counterparts (Table 1), confirming the robustness of freeze-drying in retaining extractable, thermolabile compounds. In contrast, the pilot-scale spray-drying process resulted in a markedly lower yield (approximately 9%) than the laboratory-scale operation (Table 1). This loss was attributed to an unintended rise in inlet temperature during operation (190 °C versus the nominal set point of 170 °C), which likely caused thermal softening of heat-sensitive constituents and resulted in visible sticking of material to the drying chamber and cyclone surfaces. Such wall deposition is widely recognized as a major contributor to yield loss in spray drying of botanical extracts.^25,26^ The observed temperature increase was not an isolated event but occurred in repeated pilot-scale runs, indicating scale-dependent heat transfer and airflow differences rather than a single process-control failure. In addition, teak leaf extracts contain low-molecular-weight sugars and phenolic compounds^9^ that can depress the glass transition temperature (Tg) of the powder. Materials with low Tg are prone to stickiness and wall deposition when drying temperatures exceed the sticky-point threshold,^27^ particularly at larger scales where residence time and thermal gradients differ from laboratory systems. Thus, the reduced pilot-scale yield likely reflects the combined effects of thermal variability and the intrinsic low-Tg characteristics of the extract. Regardless of the drying method, young leaf extracts exhibited higher yields than mature leaves in both scales, consistent with reports that younger plant tissues contain greater amounts of soluble polyphenols and extractable constituents.^28^

**Table 1:**
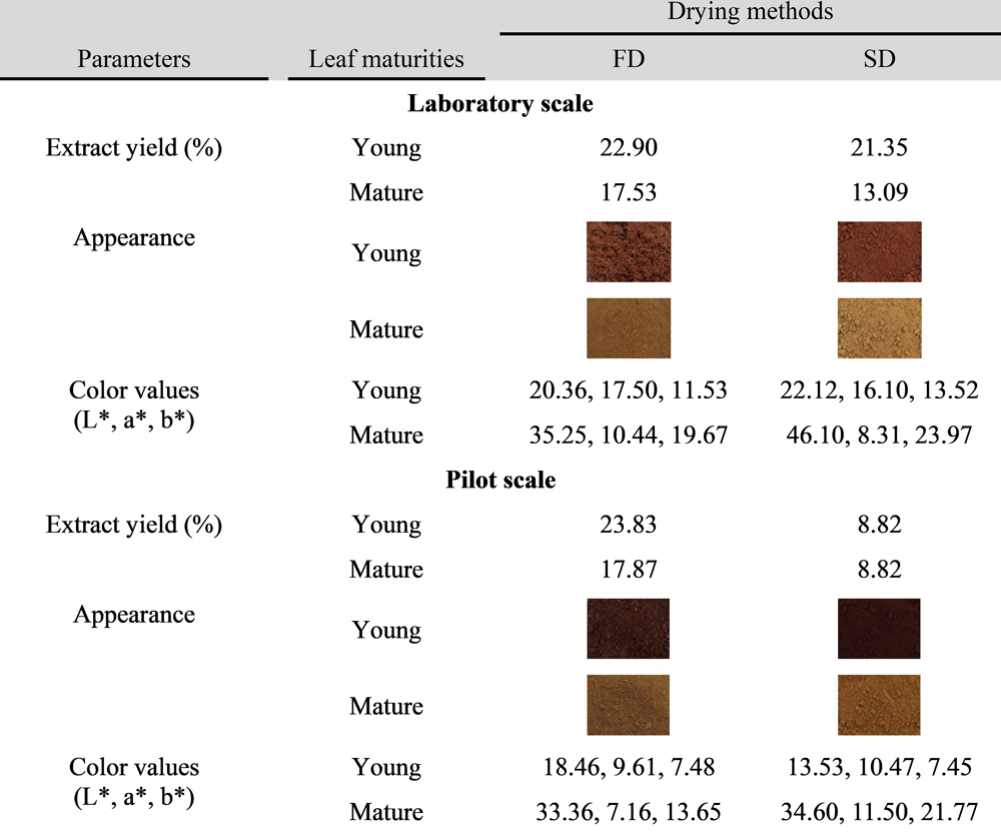
The Effect of Production Scale, Drying Method and Leaf Maturity on the Extraction Yield, Appearance and Color Shade of Teak Leaf Extracts

Color characteristics further differentiated the effects of the drying method and scale. At the laboratory scale, FD and SD young leaf extracts appeared reddish-brown, whereas mature leaf extracts were brown, with SD powders showing lighter and more yellow hues (higher L* and b* values) (Table 1) due to partial pigment degradation during spray drying.^29,30^ These findings align with our previous observations that young teak leaves produce redder extracts.^9^ At the pilot scale, SD mature leaf extracts again displayed lighter, more yellow coloration, while both FD and SD young leaf extracts darkened markedly to nearly black shades (Table 1), indicating intensified pigment degradation during scale-up extraction.

### 3.2 The Effect of Production Scale, Drying Method and Leaf Maturity on the Total Flavonoid Content (TFC), Total Phenolic Content (TPC), and Antioxidant Activity of Teak Leaf Extracts

The total flavonoid content (TFC), total phenolic content (TPC), and antioxidant activity of teak leaf extracts were strongly influenced by both the drying method and the leaf maturity. Across all conditions, freeze-drying (FD) consistently retained higher levels of TFC and TPC than spray drying (SD), resulting in lower IC_50_ values and stronger antioxidant activity (Table 2). These trends were consistent across both laboratory- and pilot-scale processing, reinforcing the effectiveness of freeze-drying in preserving thermolabile bioactive compounds.

**Table 2:** The Effect of Production Scale, Drying Method and Leaf Maturity on the Total Flavonoid Content (TFC), Total Phenolic Content (TPC), and Antioxidant Activity (IC_50_) of Teak Leaf Extracts[Table-fn tbl2fn1]

Parameters	Leaf maturities	Drying methods	
		FD	SD
**Laboratory scale**			
TFC (mg RU/g extract)	Young	16.94 ± 0.09^c^	15.15 ± 0.26^d^
	Mature	38.40 ± 0.15^a^	35.02 ± 0.36^b^
TPC (mg GAE/g extract)	Young	103.81 ± 0.98^a^	83.46 ± 0.52^b^
	Mature	68.96 ± 0.89^c^	57.64 ± 1.85^d^
DPPH IC_50_ (μg/mL)	Young	19.93 ± 0.73^a^	25.71 ± 0.86^b^
	Mature	31.95 ± 1.29^c^	42.61 ± 1.44^d^
ABTS IC_50_ (μg/mL)	Young	20.00 ± 0.45^a^	25.07 ± 0.08^b^
	Mature	40.16 ± 1.13^c^	60.97 ± 1.11^d^
**Pilot scale**			
TFC (mg RU/g extract)	Young	23.65 ± 0.30^c^	22.75 ± 0.09^d^
	Mature	41.68 ± 0.26^a^	40.39 ± 0.22^b^
TPC (mg GAE/g extract)	Young	69.41 ± 1.59^c^	62.91 ± 0.96^d^
	Mature	89.50 ± 0.34^a^	85.67 ± 1.80^b^
DPPH IC_50_ (μg/mL)	Young	30.60 ± 0.52^c^	34.20 ± 0.19^d^
	Mature	20.90 ± 0.20^a^	22.08 ± 0.57^b^
	Trolox (Positive control)	4.10 ± 0.03	
	Ascorbic acid (Positive control)	8.04 ± 0.27	
ABTS IC_50_ (μg/mL)	Young	40.14 ± 0.94^c^	53.10 ± 0.61^d^
	Mature	23.71 ± 0.29^a^	24.58 ± 0.22^b^
	Trolox (Positive control)	4.97 ± 0.10	
	Ascorbic acid (Positive control)	11.24 ± 0.45	

i Note: Values are expressed as mean ± SD (*n* = 3). Different superscript letters (a–d) within the same parameter and scale indicate statistically significant differences (*p* < 0.05) according to one-way ANOVA followed by Tukey’s multiple comparison test. Values sharing the same letter are not significantly different.

The superior retention of phenolics and flavonoids in FD samples corresponded with the higher extraction yields observed in FD extracts (Table 1). However, extraction yield did not directly predict bioactive recovery. For example, at the laboratory scale, the FD young leaf extract had a yield of 22.90% with a high TPC of 103.81 mg GAE/g, whereas the SD extract, despite a comparable yield of 21.35%, contained substantially lower TPC (83.46 mg GAE/g) (Table 2). This indicates that spray drying not only reduces yield through wall deposition but also disproportionately decreases phenolic retention, likely due to thermal degradation and oxidation during high-temperature drying. These observations align with previous studies reporting superior TFC and TPC preservation in freeze-dried botanical extracts. Baltaci et al. demonstrated significantly higher bioactive content in freeze-dried rosehip extracts compared with spray-dried samples.^29^ Correspondingly, all FD teak leaf extracts exhibited stronger radical-scavenging activity than their SD counterparts, regardless of leaf maturity, reflecting the superior preservation of antioxidant constituents under mild drying conditions. The strong agreement between DPPH and ABTS results indicates that scale-up processing and drying methods influence both hydrogen atom transfer and electron transfer-based antioxidant mechanisms. Notably, the DPPH IC_50_ values of all extracts ranged from 19.93 to 42.61 μg/mL, which are approximately 4–10 times lower than those of the reference antioxidants Trolox and ascorbic acid (Table 2). According to the classification proposed by Phongpaichit et al. (2007),^31^ these values indicate strong antioxidant activity, underscoring the high intrinsic antioxidant potential of teak leaf extracts. The consistently low IC_50_ values across drying methods further suggest that, although spray drying partially reduced phenolic.

At the pilot scale, FD extracts achieved yields comparable to those obtained at the laboratory scale (Table 1). In contrast, both FD and SD extracts exhibited substantially higher total TPC and TFC than their laboratory-scale counterparts (Table 2), indicating improved extraction efficiency upon scale-up. This enhancement can be attributed to changes in extraction hydrodynamics, as the 2 L flat-bottom flask used at the laboratory scale was replaced by a 50 L triple-wall glass reactor equipped with a cross-beam stirrer. The resulting radial flow and improved solvent distribution enhance solid–liquid contact, reduce concentration gradients, and promote mass transfer, facilitating the release of bound bioactive compounds. Continuous agitation at the pilot scale likely also improved heat transfer, further accelerating solute diffusion, consistent with reports by Zainol (2023), who observed increased TPC and TFC with higher agitation speeds.^32^

However, the intensified mass and heat transfer during scale-up may also impose greater thermal and oxidative stress on labile phenolic pigments, resulting in partial degradation. As shown in Table 2, the TPC of young leaf extracts decreased sharply at the pilot scale, reaching 69.41 ± 1.59 mg GAE/g for FD extracts and 62.91 ± 0.96 mg GAE/g for SD extracts. This pronounced decline indicates that phenolics in young leaves are particularly sensitive to large-scale processing conditions. The concurrent darkening of young leaf extracts to an almost black coloration (Table 1) further supports extensive pigment degradation and phenolic oxidation under enhanced heat transfer. Notably, this reduction in TPC was accompanied by a relative weakening of antioxidant activity, highlighting the close dependence of radical-scavenging capacity on phenolic integrity. Together, these results emphasize that while scale-up improves extraction efficiency, it can compromise the stability of heat- and oxidation-sensitive antioxidants. Therefore, further chromatographic profiling, such as Liquid Chromatography–Mass Spectrometry (LC-MS) analysis, is warranted to define acceptable compositional ranges and guide process optimization for quality specifications in large-scale production.

Chromatographic profiling is widely applied to identify phytochemicals associated with biological activities and to ensure qualitative characterization of plant extracts processed under different drying conditions.^33^ Accordingly, LC–MS analysis was performed on freeze-dried extracts to clarify compositional changes during scale-up. The chromatograms (Figure S1) revealed three major phenolic/flavonoid compounds, namely epigallocatechin 3-caffeate, delphinidin 3-O-diglucoside, and afzelechin, which exhibited markedly lower signal intensities in pilot-scale extracts compared with laboratory-scale samples (Figure S1). These compounds are well-documented as potent antioxidants^34–36^ and are therefore considered key contributors to the antioxidant activity of the extracts. Epigallocatechin 3-caffeate and afzelechin, both flavan-3-ol derivatives rich in hydroxyl groups, contribute substantially to antioxidant activity via hydrogen atom donation and electron transfer mechanisms.^37,38^ Delphinidin 3-O-diglucoside, an anthocyanin-type pigment, also contributes significantly to antioxidant activity but is known to be thermally unstable due to the instability of the flavylium cation,^35^ which can undergo hydration, ring-opening, and glycosidic cleavage under elevated temperature or prolonged processing.^39^ The reduced signal intensities of these three compounds in the pilot-scale extract suggest partial thermal and/or oxidative degradation during scale-up. This compositional change is consistent with the observed decrease in TPC and the reduced antioxidant activity, as reflected by the higher IC_50_ values for both DPPH and ABTS assays (Table 2). These findings indicate that these specific polyphenolic constituents play a significant role in the antioxidant activity and may serve as sensitive markers for monitoring quality changes during scale-up.

Leaf maturity significantly influenced the bioactive composition of the teak leaf extracts. Young leaves showed higher total TPC but lower TFC than mature leaves, regardless of the drying method. The FD young leaf extract exhibited the highest phenolic retention (103.81 mg of GAE/g; Table 2), consistent with reports that early leaf stages are enriched in phenolic acids.^40^ In contrast, flavonoid content increased with maturity, reflecting enhanced flavonol biosynthesis in mature foliage.^28,41,42^ Despite higher TFC, mature leaf extracts showed weaker antioxidant activity (higher IC_50_ values), indicating that phenolics predominant in young leaves contribute more effectively to radical scavenging. Similar maturity-dependent trends have been reported in other plant species, where young leaves display higher phenolic abundance and stronger antioxidant capacity.^43–45^

### 3.3 The Effect of Production Scale, Drying Method and Leaf Maturity on the Moisture Content and Flowing Ability of Teak Leaf Extracts

Powder moisture content and flowability are critical attributes governing the stability, handling, and end-use performance of plant extract powders. In this study, teak leaf extracts were successfully converted into powders using both freeze-drying (FD) and spray-drying (SD), with each method imparting distinct physicochemical characteristics.

At the laboratory scale, both FD and SD powders exhibited low moisture contents, ranging from 3.08% to 4.25% (Table 3), which fall within the recommended threshold for stable powder products (<5%).^46^ The slightly lower moisture levels observed in SD powders can be attributed to the elevated inlet temperature employed during spray drying, which enhances heat transfer and promotes rapid moisture removal.^47^ This behavior is consistent with previous reports indicating that SD generally produces powders with lower residual moisture due to higher drying rates.^29,48,49^ Such low moisture contents suggest that the teak leaf extract powders produced by either method are suitable for storage and further processing. At the pilot scale, residual moisture increased to approximately 6–6.5% in FD powders and 4.3–5% in SD powders (Table 3), accompanied by reduced flowability (Table 3). This increase is likely linked to enhanced recovery of hygroscopic constituents, particularly flavonoid glycosides previously identified in teak leaf extracts,^9^ whose multiple hydroxyl groups facilitate hydrogen bonding with water, thereby promoting moisture retention and increased cohesiveness in the resulting powders.

**Table 3:** The Effect of Production Scale, Drying Method and Leaf Maturity on the Moisture Content and Flowing Ability of Teak Leaf Extracts[Table-fn tbl3fn1]

Parameters	Leaf maturities	Drying methods	
		FD	SD
**Laboratory scale**			
% Moisture content	Young	3.86 ± 0.06^c^	3.08 ± 0.05^d^
	Mature	4.25 ± 0.02^a^	3.96 ± 0.07^b^
Flowing ability (Angle of repose °)	Young	35.15 ± 1.46^b^	34.38 ± 0.78^b^
	Mature	42.48 ± 0.83^a^	34.57 ± 1.34^b^
Flow property	Young	Good	Good
	Mature	Passable-may hang up	Good
**Pilot scale**			
% Moisture content	Young	5.99 ± 0.09^b^	4.30 ± 0.03^d^
	Mature	6.47 ± 0.07^a^	5.09 ± 0.11^c^
Flowing ability (Angle of repose °)	Young	45.20 ± 0.37^b^	40.76 ± 0.50^d^
	Mature	46.53 ± 1.10^a^	44.76 ± 0.90^c^
Flow property	Young	Passable-may hang up	Passable-may hang up
	Mature	Poor	Passable-may hang up

i Note: Values are expressed as mean ± SD (*n* = 3). Different superscript letters (a–d) within the same parameter and scale indicate statistically significant differences (*p* < 0.05) according to one-way ANOVA followed by Tukey’s multiple comparison test. Values sharing the same letter are not significantly different.

Leaf maturity further influenced the powder properties. Extracts derived from mature leaves consistently retained slightly higher moisture levels than those from young leaves across both drying methods (Table 3). Similar trends have been reported in other plant materials, such as soursop leaves, where mature leaves exhibited a higher intrinsic moisture content than young leaves.^50^ These differences likely reflect compositional changes during leaf development, including increased levels of structural polysaccharides or lipophilic components that can enhance water-binding capacity during drying.

Powder flowability was closely associated with moisture content but was also strongly influenced by particle morphology. At the laboratory scale, SD powders showed angles of repose below 35°, indicative of good flow behavior, whereas FD powders exhibited more variable flowability. Notably, the FD powder from mature leaves displayed reduced flow performance despite maintaining moisture levels below 5% (Table 3), suggesting that structural features and component composition also contribute to flow behavior.

Microstructural analysis provided further insight into these observations. SEM images (Figure 2) revealed that FD powders formed porous, irregular, sheet-like structures with relatively large particle sizes (150–250 μm), reflecting the development of an open matrix during ice sublimation. This porous morphology is generally associated with rapid water penetration and faster rehydration in liquid systems due to increased surface area and capillary pathways, which can be advantageous for formulations requiring rapid dissolution, such as beverages or liquid nutraceuticals. In contrast, SD powders consisted of small spherical to pseudospherical particles (∼5 μm) with wrinkled or concave surfaces, characteristic of rapid solvent evaporation and crust formation during spray drying.^30^ These dense spray-dried particles typically exhibit slower rehydration but provide improved packing and flowability, supporting ease of handling in powdered product applications.

**Figure 2:**
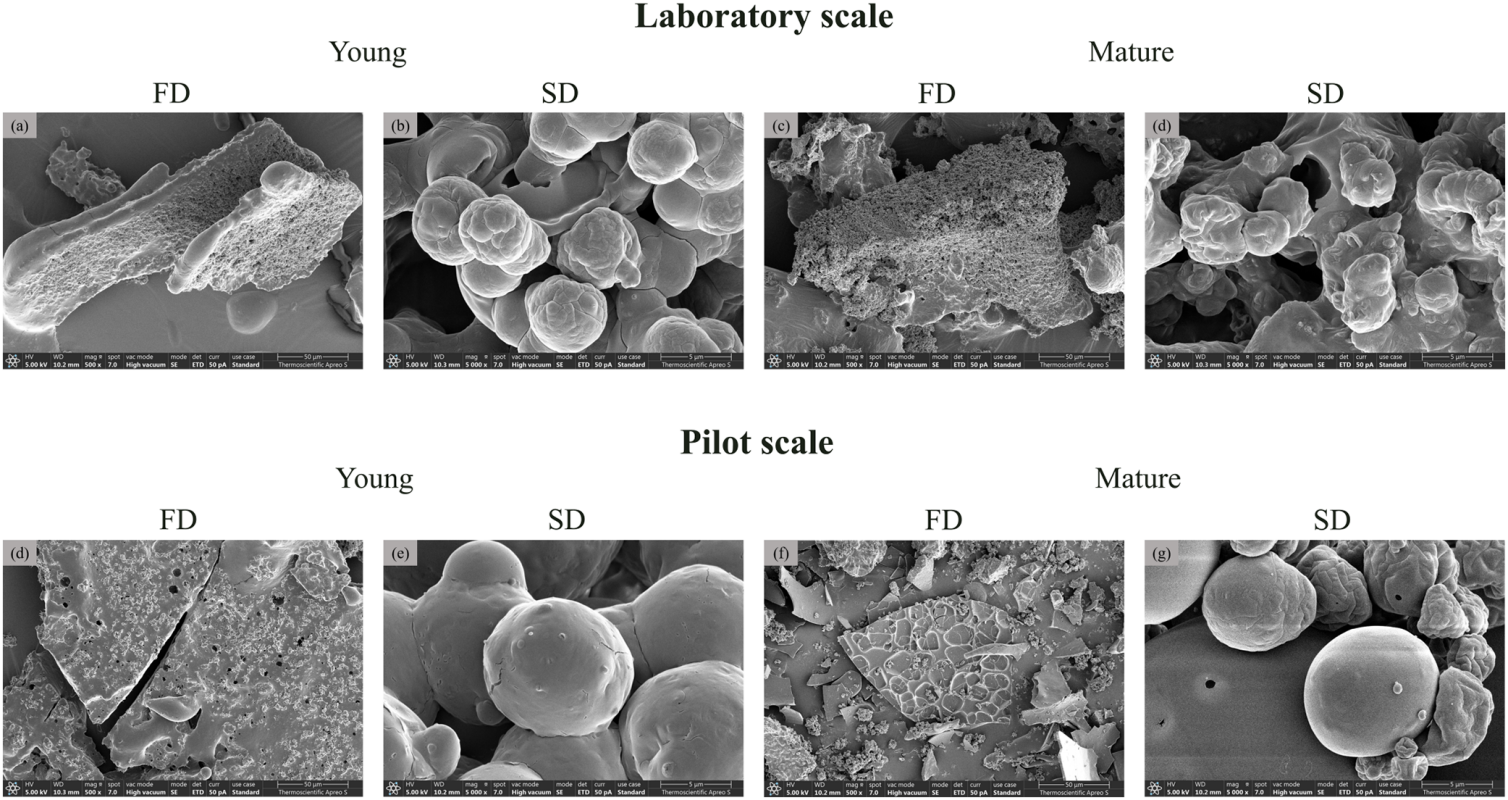
The SEM images of teak leaf extracts obtained at lab-scale are as follows: (a) young FD (500x), (b) young SD (5,000x), (c) mature FD (500x), and (d) mature SD (5,000x). At pilot scale (e) young FD (500x), (f) young SD (5,000x), (g) mature FD (500x), and (h) mature SD (5,000x).

Overall, FD produces powders with highly porous structures that may favor reconstitution and bioactive accessibility, albeit with increased moisture sensitivity and reduced flowability, particularly on a larger scale. SD, by contrast, yields powders with a compact morphology, lower residual moisture, and superior flow behavior, supporting easier handling and large-scale processing. These complementary characteristics highlight the importance of aligning the drying strategy with the intended application of teak leaf extracts, whether prioritizing functional performance or manufacturing.

### 3.4 The Effect of Production Scale, Drying Method and Leaf Maturity on the Thermal Stability of Teak Leaf Extracts

Thermal stability of teak leaf extracts was assessed by differential scanning calorimetry (DSC), revealing structural differences related to the drying method and scale. At the laboratory scale, FD powders exhibited sharper, more intense endothermic peaks than SD powders (Figure 3a), indicating higher structural uniformity and greater crystallinity. For instance, the enthalpy change (ΔH) of FD mature leaf extract was −122.84 J/g, compared with −106.36 J/g for SD (Table 4), reflecting stronger molecular interactions and a more ordered solid matrix. SD powders displayed broader peaks and lower ΔH, consistent with more amorphous structures formed under rapid solvent evaporation and thermal stress.^14,30^ Slightly higher residual moisture in FD powders (<5%) may act as a plasticizer, marginally reducing Tm,^51,52^ but their sharper peaks confirm better structural order.

**Figure 3:**
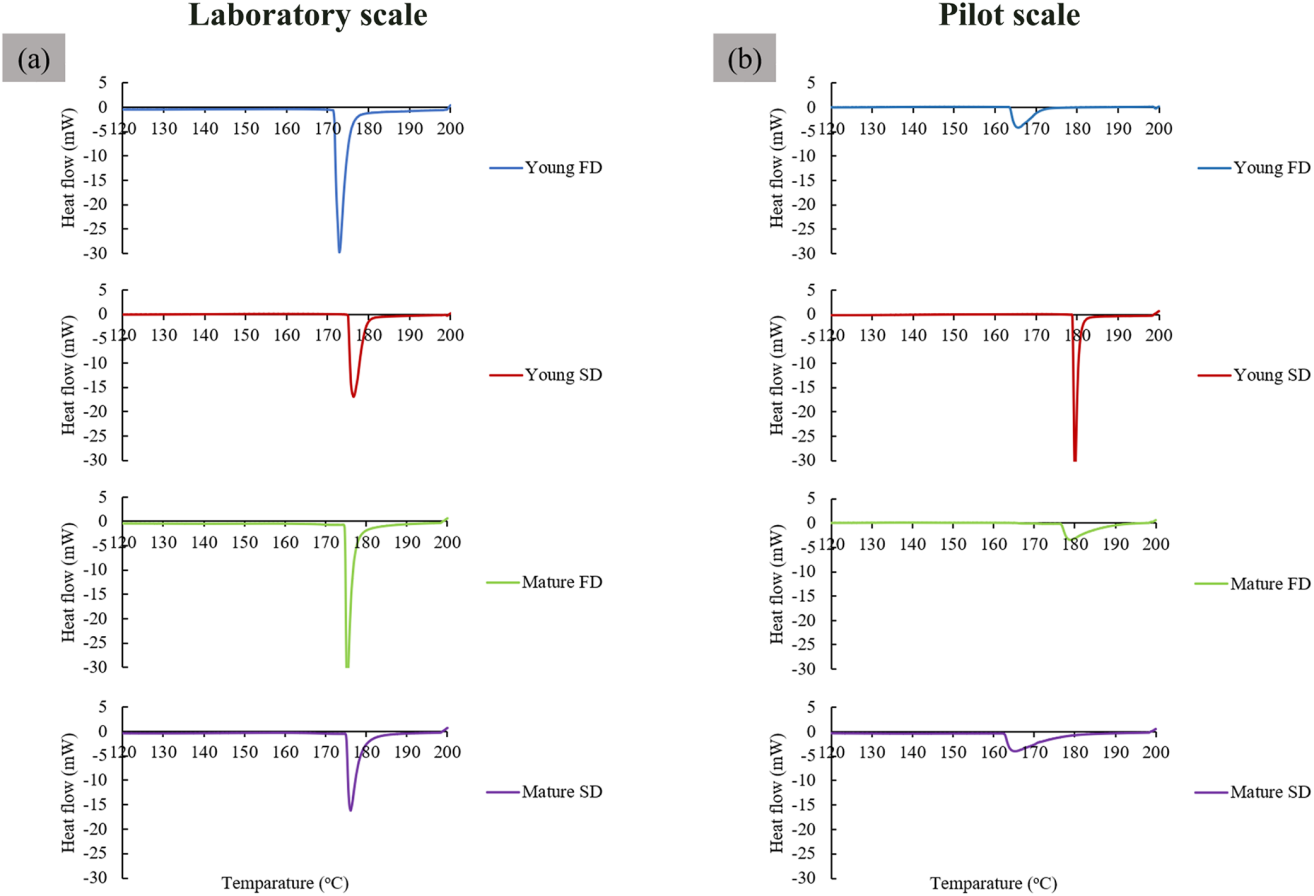
The DSC thermograms of teak leaf extracts were obtained at (a) lab scale and (b) pilot-scale.

**Table 4:** The Effect of Production Scale, Drying Method and Leaf Maturity on the Thermal Stability of Teak Leaf Extracts

Parameters	Leaf maturities	Drying methods	
		FD	SD
**Laboratory scale**			
Melting temperature (Tm; °C)	Young	171.38	174.84
	Mature	174.61	174.89
Enthalpy change (ΔH; J/g)	Young	–116.63	–115.14
	Mature	–122.84	–106.36
**Pilot scale**			
Melting temperature (Tm; °C)	Young	163.54	178.82
	Mature	176.70	162.71
Enthalpy change (ΔH; J/g)	Young	–57.75	–89.00
	Mature	–54.02	–78.98

At pilot scale, DSC thermograms for both FD and SD powders showed broader peaks, except for the SD young leaf extract, which retained a sharp peak (Figure 3b). Enthalpy values decreased to −54 to −89 J/g compared to the laboratory scale (Table 4), reflecting reduced thermal stability and less organized solid-state structures. The higher heat transfer during large-scale extraction may partially degrade sensitive phenolic compounds, contributing to less crystalline matrices the require less energy for disruption. FD powders at the pilot scale showed ΔH of −54 to −57 J/g, lower than SD (−78 to −89 J/g), indicating a more heterogeneous solid matrix with reduced energetic stability. These changes are influenced by scale-up factors, including differences in refrigeration capacity, shelf mass, heat transfer, and load configuration, which may affect sublimation rates and powder structure.^51,53–55^

From an industrial perspective, the choice of drying method also impacts energy demand and production efficiency. A comparative estimation of pilot-scale energy demand revealed substantial differences between FD and SD. The FD process comprised 24 h of prefreezing at −80 °C followed by 84 h of vacuum drying (12 h at 0 °C and 72 h at 30 °C). Based on typical equipment power ratings (approximately 1.5 kW for the freezer and 8 kW for the freeze-dryer), the total estimated energy consumption was approximately 708 kWh per 15 L batch. In contrast, SD operated at a feed rate of 0.75 L/h, requiring approximately 20 h to process the same volume. With an average power draw of 18 kW, total energy consumption was estimated at approximately 360 kWh per batch. Thus, FD required nearly twice the total energy input and more than five times longer processing time than SD. Although FD better preserved phenolic and flavonoid compounds, SD demonstrated clear advantages in throughput and batch-level energy efficiency.

In summary, the drying method and leaf maturity jointly governed the physicochemical and bioactive properties of teak leaf extracts. Freeze-drying favored molecular organization, phenolic and flavonoid retention, and antioxidant activity, although the resulting powders showed higher moisture content and reduced flowability, particularly at larger scales. In contrast, spray-dried powders exhibited lower moisture, more uniform particle morphology, and improved handling, albeit with moderately reduced retention of sensitive compounds. Leaf maturity further shaped extract composition, with young leaves being richer in phenolics and having stronger antioxidant activity, while mature leaves contained higher flavonoid levels.

Notably, the teak leaf extracts produced in this study exhibited substantially higher total phenolic contents than previously reported (6.17–46.12 mg GAE/g),^12,31^ underscoring their strong antioxidant potential. These compositional and physical distinctions support application-specific use across food, cosmetic, and pharmaceutical products: freeze-dried extracts are well-suited for functional foods, nutraceuticals, and antioxidant-enriched cosmetic formulations, particularly in liquid or reconstituted systems, whereas spray-dried extracts are more suitable for powdered products such as instant beverages, supplements, and cosmetic powders, where flowability and dispersibility are critical. Collectively, these findings highlight the versatility of teak leaf extracts and their potential for tailored.

## 4 Conclusions

This study elucidates how drying techniques and production scale collectively shape the physicochemical properties, structural characteristics, and functional performance of teak leaf extracts, enabling the valorization of an abundant agricultural byproduct into a high-value bioresource. The drying method applied proved to be the primary determinant of extract quality, with freeze-drying (FD) and spray drying (SD) producing powders with distinct solid-state structures and attributes that directly reflect drying-induced differences in microstructure, moisture retention, and molecular organization. FD favored higher extract yields, superior preservation of phenolic and flavonoid compounds, and stronger antioxidant activity. DSC analysis further confirmed that FD generated more ordered and energetically stable matrices, indicating enhanced molecular integrity under mild, low-temperature drying conditions.

The choice of drying technique equally governed powder functionality. Spray drying generated powders with lower residual moisture, spherical particle morphology, and improved flowability, attributes advantageous for large-scale manufacturing and powder handling, though at the cost of lower ΔH values, consistent with more amorphous structures and partial loss of molecular order attributable to thermal exposure. At the pilot scale, the trends observed at the laboratory scale were retained for both drying methods, demonstrating their robustness, while yield losses during SD underscored the sensitivity of phytochemicals to heat and the importance of careful process optimization during scale-up.

Overall, the findings highlight the complementary strengths of each drying technique, where FD supports bioactive preservation and antioxidant functionality, while SD offers superior powder processability and scalability. This work advances the understanding of structure–property–processing relationships in plant extract drying and demonstrates the suitability of teak leaf extract, valorized from forestry and agricultural waste, as a sustainable natural colorant and antioxidant for food, cosmetic, and pharmaceutical applications, supporting environmentally responsible production strategies.

## Supplementary Material



## Data Availability

The data are available from the corresponding author upon reasonable request.

## Notes

LC-MS profile and chemical structure of compound substances in the young FD extract from teak leaves in both laboratory-scale (red line) and pilot-scale (black line) (PDF)

## Abbreviations

FD: freeze-drying
SD: spray drying
Na_2_CO_3_: sodium carbonate
AlCl_3_ 6H_2_O: aluminum chloride hexahydrate
TFC: total flavonoid content
TPC: total phenolic content
AA: antioxidant activity
Tg: glass transition temperature
Tm: melting temperature
DSC: differential scanning calorimetry
SEM: Scanning Electron Microscope
