# Supplementary material for: Valorization of Teak Leaf Agricultural Waste: Impact of Drying Technique and Production Scale on Extract Quality
Source: ACS Omega. 2026 Jun 11;11(24):35315–25. doi: 10.1021/acsomega.6c00281 (PMC13295025; doi:10.1021/acsomega.6c00281)
Supplement: Supplementary file 1 [file ao6c00281_si_001.pdf]

SUPPORTING INFORMATION

Valorization of Teak Leaf Agricultural Waste: Impact of Drying Technique and Production Scale on Extract Quality

*Natthawadee Tibkawin[1], Nichapa Buasumrit[1], Panatpong Boonnoun[2], Sukunya Ross[3], Gareth Ross[3], Jarupa Viyoch[1], Pensri Charoensit[1]**

[1]Department of Pharmaceutical Technology, Faculty of Pharmaceutical Sciences and Center of Excellence for Innovation in Chemistry, Naresuan University, Phitsanulok 65000, Thailand

[2]Department of Industrial Engineering, Chemical Engineering Program, Faculty of Engineering, Naresuan University, Phitsanulok, 65000, Thailand

[3]Department of Chemistry, Center of Excellence in Biomaterials, Faculty of Science, Naresuan University, Phitsanulok 65000, Thailand

Supporting Result

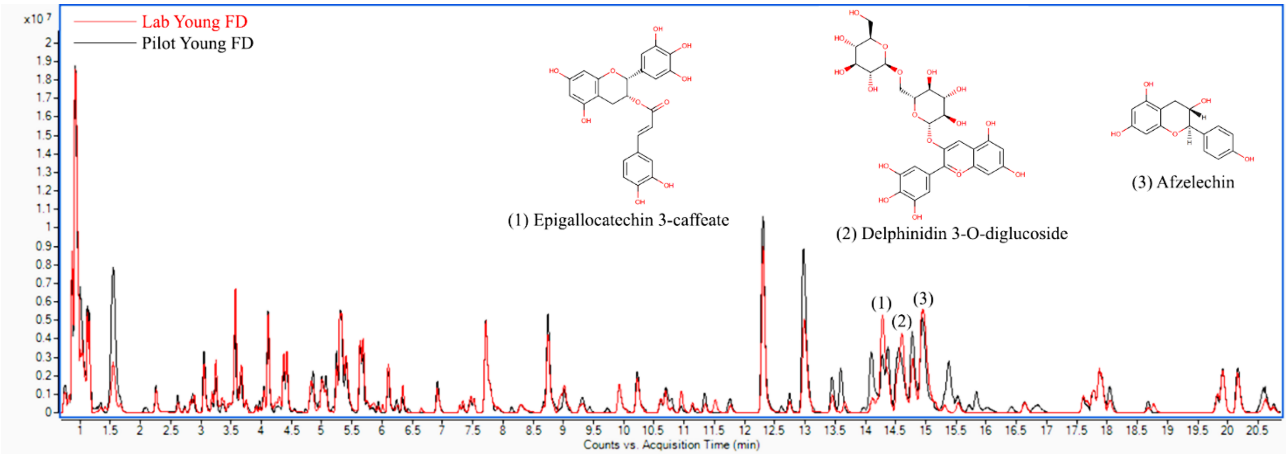

Figure S1 LC-MS profile and chemical structure of compound substances in the young FD extract from teak leaves in both laboratory-scale (red line) and pilot-scale (black line).

Supporting Experimental (For Figure S1)

Liquid chromatography-mass spectrometry (LC-MS) analysis. LC-MS analysis was performed to identify the compounds of the young FD teak leaf extracts in both laboratory- and pilot-scales, using an Agilent 1260 infinity high-performance liquid chromatography instrument connected to an Agilent G6540B MS Q−TOF equipped with an electrospray ionization (ESI) source in negative ion mode. Fragmentations were performed using auto MS/MS experiments with collision energies at 10, 20 and 40 V. Separation was carried out with a Phenomenex Luna C−18(2) column (5 µm, 150 × 4.6 mm) with the gradient mobile phase comprising 0.1% v/v formic acid in purified water (solvent A) and 0.1% v/v formic acid in acetonitrile (solvent B). Both the young FD teak leaf extracts in laboratory- and pilot-scales were prepared at a concentration of 20 mg/mL.
